# Decoding the relationship between ageing and amyotrophic lateral sclerosis: a cellular perspective

**DOI:** 10.1093/brain/awz360

**Published:** 2019-12-18

**Authors:** Virenkumar A Pandya, Rickie Patani

**Affiliations:** 1 Department of Neuromuscular Diseases, University College London Queen Square Institute of Neurology, Queen Square, London, UK; 2 The Francis Crick Institute, London, UK

**Keywords:** amyotrophic lateral sclerosis, ageing, neuromuscular junction, lower motor unit, healthspan

## Abstract

With an ageing population comes an inevitable increase in the prevalence of age-associated neurodegenerative diseases, such as amyotrophic lateral sclerosis (ALS), a relentlessly progressive and universally fatal disease characterized by the degeneration of upper and lower motor neurons within the brain and spinal cord. Indeed, the physiological process of ageing causes a variety of molecular and cellular phenotypes. With dysfunction at the neuromuscular junction implicated as a key pathological mechanism in ALS, and each lower motor unit cell type vulnerable to its own set of age-related phenotypes, the effects of ageing might in fact prove a prerequisite to ALS, rendering the cells susceptible to disease-specific mechanisms. Moreover, we discuss evidence for overlap between age and ALS-associated hallmarks, potentially implicating cell type-specific ageing as a key contributor to this multifactorial and complex disease. With a dearth of disease-modifying therapy currently available for ALS patients and a substantial failure in bench to bedside translation of other potential therapies, the unification of research in ageing and ALS requires high fidelity models to better recapitulate age-related human disease and will ultimately yield more reliable candidate therapeutics for patients, with the aim of enhancing healthspan and life expectancy.

## Teaser

Ageing is the biggest risk factor for ALS. Pandya and Patani review cell type-specific molecular links between ageing and ALS, focusing on the lower motor unit (motor neurons, skeletal muscle, astrocytes and Schwann cells), and consider how cell type-specific ageing mechanisms could serve as therapeutic targets.

## Introduction

**Figure 1:**
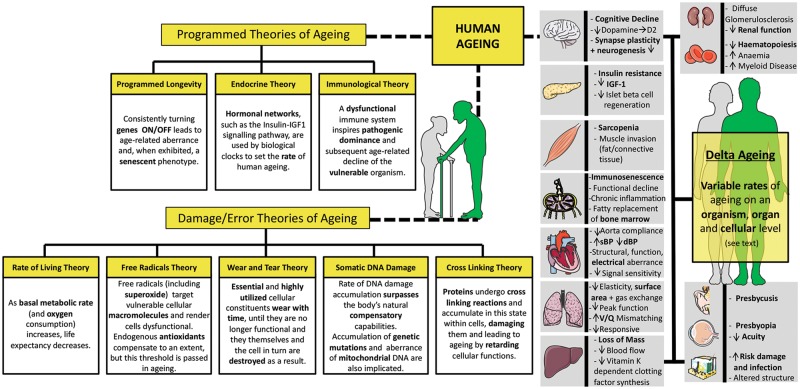
**Human ageing theories and phenotypes.** A number of theories aim to explain human ageing (reviewed in Jin, 2010), broadly categorized into the programmed theories of ageing, where normal ageing follows a set biological clock with time-dependent expression changes, and damage theories of ageing, where accumulation of damage over time ultimately leads to dysfunction (reviewed in Jin, 2010). Age-related abnormalities (described above) are apparent in several organs (reviewed in Khan *et al.*, 2017); however, differential resistance/vulnerability to the effects of ageing in various organs has been noted (reviewed in Khan *et al.*, 2017). The rate of ageing differs between individuals, with some people ageing better and some worse than expected in a phenomenon termed Delta ageing (Rhinn and Abeliovich, 2017). Indeed, variability of ageing rate might also occur on a cellular and organ level, somewhat providing evidence for the mechanism behind cell type and organ specific susceptibility to the effects of ageing, and in turn age-related disease, such as ALS. Templates used/adapted to create this figure are freely available from Servier Medical Art (https://smart.servier.com/).

The human population is ageing, with an estimated 1.5 billion people expected to be 65+ years by 2050, triple the 2010 estimate (World Health Organisation, 2011). But alongside a lengthened life expectancy comes the drawback of age-related ill health that compromises quality of life. Ageing is a ubiquitous phenomenon, with multiple hypotheses attempting to explain why age-related changes occur on an organism, organ and cellular level (reviewed in Jin, 2010; Lopez-Otin *et al.*, 2013) (Fig. 1). Indeed, age is the most prevalent risk factor for neurodegenerative disease (reviewed in Khan *et al.*, 2017). Within this group is amyotrophic lateral sclerosis (ALS), a relentlessly progressive and universally fatal disease underpinned by degeneration of motor neurons. With a prognosis of 2–5 years from onset to fatality and a myriad of complex debilitating symptoms (reviewed in Balendra and Patani, 2016), it is key to elucidate the true pathogenic mechanisms underlying ALS and use these insights to develop truly impactful disease-modifying therapies for patients, a feat yet to be achieved.

**Figure 2:**
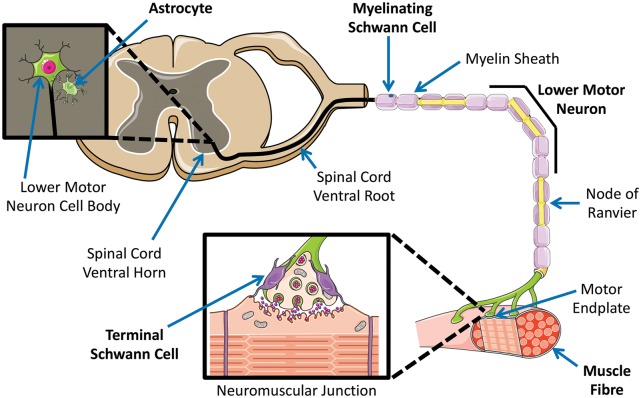
**The lower motor unit.** Individual components of the lower motor unit: lower motor neuron, skeletal muscle, astrocyte, myelinating Schwann cell, terminal Schwann cell. All constituents of the lower motor unit play key roles in motor function and voluntary movement, are affected by normal ageing and are implicated in ALS pathogenesis. The site of unification of motor neuron and muscle (the neuromuscular junction) has a vital role in ALS pathology and also undergoes age-associated alterations. Templates used/adapted to create this figure are freely available from Servier Medical Art (https://smart.servier.com/).

Several studies have implicated the neuromuscular junction (NMJ), the site of union between motor neuron and muscle within the lower motor unit (Fig. 2), in ALS pathogenesis. Indeed, the die-back hypothesis of ALS suggests that motor neuron terminals at the NMJ are the initial foci of pathogenesis with retrograde axonal degeneration ultimately reaching the motor neuron soma, leading to neuronal degeneration and subsequent symptoms (reviewed in Dadon-Nachum *et al.*, 2011). Neuromuscular transmission defects and synaptic aberrance have been shown to precede motor neuron degeneration and motor symptoms in rodent (Rocha *et al.*, 2013; Chand *et al.*, 2018) and fruit fly (Shahidullah *et al.*, 2013) models of ALS. Furthermore, restricting expression of ALS-associated human superoxide dismutase 1 (SOD1) to skeletal muscle, induced motor neuron degeneration and functional defects in transgenic mice overexpressing wild-type human SOD1 or its G93A and G37R mutant forms (Wong and Martin, 2010). This, alongside findings of altered regulation of skeletal muscle specific microRNAs in ALS (reviewed in Di Pietro *et al.*, 2018), fortifies the role of skeletal muscle and the NMJ in ALS pathology, whilst supporting the die-back hypothesis.

Here, we review how ageing of the cellular constituents of the lower motor unit relates to ALS. Specifically, we will discuss motor neurons, skeletal muscle, astrocytes and Schwann cells. By integrating insights from these individual components, we discuss the potential role of cell type specific ageing in ALS. Finally, we look at approaches to enhance ALS model fidelity and applicability to patients, as well as potential therapeutic implications of tackling age-associated aberrance, namely maximizing healthspan and lifespan in ALS.

### Ageing of the motor neuron

The degeneration of brain and spinal cord motor neurons forms the major pathological substrate of ALS, leading to rapid functional decline and death in patients. As well as the clear contribution of non-neuronal cells to ALS, a number of cell intrinsic motor neuronal pathological hallmarks have been defined, including (but not restricted to) excitotoxicity, abnormal cytoskeleton and axonal transport and disrupted RNA metabolism (reviewed in Van Damme *et al.*, 2017). Indeed, normal ageing bears a variety of structural and functional consequences for motor neurons, which may directly or indirectly contribute to motor neuron pathology in ALS.

Age-related changes in motor neuron number remains a controversial topic, with some studies suggesting motor neuron number and/or size to be stable with ageing in mice and rhesus monkeys (Maxwell *et al.*, 2018), whilst other studies suggest progressive motor neuronal loss [in rat (Jacob, 1998) and human (Tomlinson and Irving, 1977) lumbosacral spinal cords]. Indeed, neither the aged rats nor patients from these studies experienced commensurate loss of physical activity/ability as a result of motor neuron attrition (Tomlinson and Irving, 1977; Jacob, 1998), suggesting a significant functional reserve in this system. Despite not causing outright functional decline, it remains possible however that a reduction in motor neurons with ageing leaves remaining aged motor neurons under elevated stress (Jacob, 1998), and thereby more vulnerable to age-related pathologies, such as ALS.

Voluntary movements depend on effective electrical communication between neurons, with imperative roles for both excitatory (glutamatergic and cholinergic) and inhibitory (GABAergic and glycinergic) synaptic inputs terminating on alpha motor neurons (Maxwell *et al.*, 2018). Indeed, cholinergic synaptic inputs in the ventral horn and specifically those terminating on alpha motor neuron cell bodies were decreased in old rhesus monkeys, a finding mirrored in mice (Maxwell *et al.*, 2018). Glutamatergic synaptic inputs directly terminating on alpha motor neurons in old monkeys and mice were also reduced (Maxwell *et al.*, 2018). Hence, normal ageing is accompanied by loss of synaptic inputs to alpha motor neurons, a key age-related phenotype and indeed, a shared pathological hallmark with motor diseases including ALS [as shown in transactive response DNA binding protein 43kDa (TDP-43) and SOD1 mutant mice] (Vaughan *et al.*, 2015).

Neurons are post-mitotic, meaning they have left the cell cycle and are no longer proliferating, thereby they cannot undergo classical cellular senescence. Emerging literature has however implicated an analogous process in neurons, mimicking some of the key age-related effects of senescence on other cell types. More specifically, human induced pluripotent stem cell (iPSC)-derived neurons from patients with Rett syndrome, characterized by loss-of-function mutations in MECP2, were shown to activate p53, a regulator of cellular senescence, subsequently inhibiting complex neuronal process formation (Ohashi *et al.*, 2018). In addition, senescence-associated secretory phenotype (SASP) genes were also induced and β-galactosidase activity increased in neurons lacking MECP2 (Ohashi *et al.*, 2018), indicating that a ‘senescence like’ picture was present in neurons derived from these patients. It is possible that an analogous senescence process takes place in normal ageing neurons, thus leading to cellular stress, aberrant neuronal health and enhanced vulnerability to further pathological insult.

Lipofuscin aggregates, rich in lipids, metals and misfolded proteins, accumulate in neurons during normal ageing, as well as in other post-mitotic, non-proliferative cell types that lack the capacity to effectively dilute out the aggregates during proliferation (reviewed in Moreno-Garcia *et al.*, 2018). Indeed, lysosomes and subsequently cell cytoplasm become overloaded with these aggregates, with associated oxidative stress, altered proteostasis, neuronal cytoskeletal and trafficking perturbations, and glial reactive transformation, potentially modifying risk of neurodegenerative disease (reviewed in Moreno-Garcia *et al.*, 2018). Given that lipofuscin aggregate accumulation has been consistently noted in various aged animal (Maxwell *et al.*, 2018) and indeed human motor neurons during normal ageing (Tomlinson and Irving, 1977; Rygiel *et al.*, 2014), this phenomenon may thereby be relevant in ALS.

The dysfunction of motor neuron mitochondria with normal ageing (Rygiel *et al.*, 2014) is intriguing, seeing that this mechanism has been noted as a key contributor to ALS pathology (reviewed in Van Damme *et al.*, 2017). Lumbar spinal cord sections from 12 elderly patients revealed a subset of motor neurons with mitochondrial respiratory chain complex 1 deficiency, a phenotype not present in human foetal (9–11 weeks post-conception) spinal cords (Rygiel *et al.*, 2014). Mitochondrial DNA copy number and cell body size were also reduced in complex 1 deficient motor neurons (Rygiel *et al.*, 2014). With potential effects on neuronal function, viability and survival, it is possible that respiratory chain deficiency with normal ageing may instigate motor neuron dysfunction and degeneration (Rygiel *et al.*, 2014) and this is consistent with such defects having an important role in age-related neurodegeneration and ALS, although this clearly requires further direct investigation to understand comprehensively.

Electrophysiological studies on aged wild-type mice showed alterations in motor neuron membrane and excitability properties (Moldovan *et al.*, 2016). Indeed, ageing led to changes in voltage gated sodium channel expression, more specifically, ectopic expression of Na_v_1.8 on aged motor axons, affecting axonal membrane functionality (Moldovan *et al.*, 2016). These electrophysiological alterations were attenuated with pharmacological blocking of Na_v_1.8, and in sensory neuron-specific Na_v_1.8 null mice (Moldovan *et al.*, 2016). Altogether, although itself not neurotoxic, ectopic expression of Na_v_1.8 during ageing can leave motor neurons with higher energy requirements vulnerable to progression of neurodegeneration and neuronal pathology (Moldovan *et al.*, 2016). Age-related membrane excitability alterations and changes potentially consistent with membrane depolarization were also noted in a non-invasive electrophysiological study of patient median motor axons (Bae *et al.*, 2008). Age-associated electrical abnormalities may thereby leave aged motor neurons susceptible to further neuronal insult and neurodegenerative pathology.

A number of studies have identified key genes and pathways in normal motor neuron ageing, which can help better understand the potential intersect between ageing and disease. Indeed, transcriptomic analysis in *Drosophila* revealed matrix metalloproteinase 1 (dMMP1) to not only undergo an age-related increase in expression in motor neurons, but also cause motor functional defects that become more severe with further ageing when overexpressed in a subset of motor neurons (Azpurua *et al.*, 2018). Impairment of presynaptic neurotransmitter release at the NMJ was the proposed mechanism (Azpurua *et al.*, 2018). The upregulation of matrix metalloproteinases in ageing may be of special significance in age-related neurodegeneration and namely ALS, with TDP-43 overexpression in neurons accelerating the rate of dMMP1 accumulation (Azpurua *et al.*, 2018) and suggesting a potential pathogenic mechanism linking ageing and ALS.

Mice with perturbed excision repair cross-complementation group 1 gene (*Ercc1*^Δ/−^ mice), deficient in a number of DNA repair system components including nucleotide excision repair and double strand break repair, gained an aberrant motor phenotype that progressively declined with ageing (de Waard *et al.*, 2010). Alongside activation of CNS microglia and astrocytes, age-associated motor neurodegeneration and NMJ pathology, genotoxic stress, DNA damage and Golgi apparatus abnormalities were noted in *Ercc1*^Δ/−^ mice (de Waard *et al.*, 2010). Hence, defective DNA repair mechanisms lead to motor neuron degeneration and functional decline in an age-dependent manner (de Waard *et al.*, 2010). TDP-43 and fused in sarcoma (FUS) pathology did not develop in these motor neurons, suggesting DNA damage from ERCC1 deficiency is not sufficient to recapitulate ALS-related pathology (de Waard *et al.*, 2010). Nonetheless, DNA damage accumulation with normal ageing can prove a vital risk factor contributing to neurodegenerative disease and ALS (de Waard *et al.*, 2010).

Despite not causing motor functional decline, transgenic expression of mutant heat shock protein beta 1 (HSPB1), associated with motor neuropathies, showed age-dependent subclinical motor axonal pathology, characterized by electrophysiological changes and neuropathological hallmarks (Srivastava *et al.*, 2012). Conditional knockout of dynactin P150^Glued^ in murine neurons not only led to age-dependent motor functional decline but also caused preferential degeneration of spinal motor neurons in aged animals (Yu *et al.*, 2018). Many deleterious phenotypes only present when the animals in these studies age, which raises the hypothesis that normal ageing might be a prerequisite for motor neuronal degeneration in ALS. It is possible that the ageing of motor neurons, in addition to causing direct cellular phenotypes, might render the system vulnerable to subsequent ALS disease-specific mechanisms, although further studies are required to definitively resolve this.

With evidence suggesting that normal ageing affects motor neuron number, structure and functional capacity, it is unsurprising that age-related effects may play a vital role in neurodegenerative diseases involving motor neurons, such as ALS. An integration of ageing and ALS research can allow for better mechanistic insight and therapeutic advancement, ultimately leading to patient benefit.

### Ageing of skeletal muscle

The nervous system and skeletal muscle are intimately linked, with motor neuron-derived electrical stimulation ultimately allowing muscle contraction and, in turn, movement. As the postsynaptic constituent of the NMJ, muscle itself has been implicated as an early component in ALS pathogenesis, with muscle weakness an initial and debilitating clinical symptom (reviewed in Hobson and McDermott, 2016). Indeed, skeletal muscle-specific expression of mutant (G93A/G37R) and wild-type human SOD1 in transgenic mice disrupted NMJs and led to motor neuron degeneration and a corresponding functional phenotype (Wong and Martin, 2010). Mitochondrial dysfunction, namely alterations in morphology and distribution, and the induction of protein kinase Cθ have been implicated as key mechanisms destabilizing NMJs in transgenic mice with muscle restricted SOD1^G93A^ (Dobrowolny *et al.*, 2018). As well as its implications in ALS, skeletal muscle undergoes a variety of structural and functional changes in normal ageing, which may also link to its roles in disease. Sarcopenia, the highly prevalent, age-associated decline in skeletal muscle mass, force and function, not only significantly impacts patient quality of life, but also bears key connotations for the healthcare system owing to its links with frailty (Clegg *et al.*, 2013), falls, disability and mortality (reviewed in Marzetti *et al.*, 2017). The clinical phenotype of sarcopenia is underpinned by the effects of ageing on skeletal muscle and its environment (reviewed in Marzetti *et al.*, 2017), which we discuss below.

Skeletal muscle adult stem cells (satellite cells) reside between muscle fibre sarcolemma and basement membrane in a quiescent state, but, on injury, have the capacity to asymmetrically divide to both self-replicate and form progeny which ultimately differentiate to new muscle fibres (Morgan and Partridge, 2003). With ageing, satellite cells lose their capacity to regenerate damaged muscle (Sousa-Victor *et al.*, 2014*b*), with cell intrinsic alterations implicated.

Indeed, induction of P16^INK4a^ in geriatric mice, a regulator of cellular senescence, drove satellite cells to a pre-senescent phenotype, which was further advanced to irreversible full senescence when the cells were placed under proliferative pressure (Sousa-Victor *et al.*, 2014*b*). Functionally, the cells showed defects in activation, ability to proliferate and capacity to self-renew, altogether preventing successful muscle fibre regeneration (Sousa-Victor *et al.*, 2014*b*). Adult (5–6 months) and old (20–24 months) murine satellite cells actively repress P16^INK4a^ to maintain a state of reversible quiescence, which underpins their regenerative function. Geriatric (28–32 months) animals had P16^INK4a^ repression lifted, and underwent the abovementioned state change (reversible quiescence → irreversible pre-senescence → geroconversion to full senescence). Knocking out Bmi1, a component of the main repressor of the INK4a locus, induced a senescent-like phenotype in young satellite cells with resultant functional defects (Sousa-Victor *et al.*, 2014*a*). Interestingly, from a therapeutic perspective, inhibition of P16^INK4a^ in geriatric and progeric mouse models was sufficient to reverse the senescent phenotype and restore regeneration (Sousa-Victor *et al.*, 2014*a*). Thereby, with aged satellite cells unable to facilitate skeletal muscle recovery following insult, it may be left more vulnerable to further disease-specific pathology in ALS.

Protein arginine methyltransferase 7 (PRMT7) knockout mice showed reduced skeletal muscle mass and increased fat at 8 months of age, with delayed differentiation and premature senescence as putative underlying mechanisms. Increased p21 (senescence marker) and reduced DNMT3b were noted, with restoration of the latter rescuing the senescent phenotype *in vitro*. Although regenerative capacity was similar between young wild-type and *Prmt7*^−/−^ mice 21 days following tibialis anterior cardiotoxin injury, the knockouts showed significant structural regenerative aberrance with age (8 months) when compared to *Prmt7*^−/−^ uninjured and wild-type injured/uninjured mice. Indeed, satellite cell number, self-renewal ability and regenerative function were defective (Blanc *et al.*, 2016). Mice heterozygous for Ku80 (*Xrcc5*), a facilitator of genomic and telomere stability, showed a muscle phenotype resembling accelerated physiological ageing. Following recurrent injury, heterozygous mice (and Ku80 null mice) showed fewer self-renewing stem cells, with a corresponding increase in committed and expanding cells. Injuring the tibialis anterior muscle of adult Ku80 wild-type, heterozygous and null mice twice (15-day interval) resulted in decreased regeneration in the 18-month compared to the 2-month wild-type, as well as reduced capacity to regenerate in Ku80 heterozygous and null mice (as measured 7 days after second injury) (Didier *et al.*, 2012). The heterozygous stem cells were also shown to have significantly shorter telomeres than wild-type mice as well as features of skeletal muscle premature ageing (Didier *et al.*, 2012). Satellite cells also lose functional heterogeneity with age, whilst maintaining the clonal complexity of their youthful counterparts, as visualized using *in vivo* multicolour lineage tracing (Tierney *et al.*, 2018). Aged satellite cells obtained via muscle biopsy of sedentary elderly patients showed deficits in antioxidant activity, cell membrane fluidity and intracellular basal calcium content compared to those from newborn or sedentary young patients (Fulle *et al.*, 2005). Indeed, other intrinsic age-related satellite cell alterations might include DNA damage and mitochondrial abnormalities (reviewed in Brack and Munoz-Canoves, 2016), resembling molecular mechanisms in ALS (reviewed in Van Damme *et al.*, 2017).

Altogether, satellite cells develop a number of cell intrinsic changes with ageing, ultimately leading to their dysfunction and a homeostatically aberrant skeletal muscle system that is vulnerable to disease-specific insult. Moreover, ALS satellite cells have been shown to lose their differentiation potential (and consequently their regenerative capacity) compared to controls (Scaramozza *et al.*, 2014), indicating shared phenotypic features between aged and ALS satellite cells.

As well as the abovementioned intrinsic satellite cell alterations, the niche in which these cells reside also undergoes age-associated changes. Nuclear factor kappa-light-chain-enhancer of activated B cells (NF-κB), for example, is activated during ageing (Zhang *et al.*, 2017). Specifically increasing NF-κB signalling in satellite cells led to impaired repair following cryoinjury, a phenotype that was rescued by administration of an NF-κB inhibitor (Oh *et al.*, 2016). Isolation of satellite cells prior to injury indicated no intrinsic differences in proliferation or initiation of myogenesis. The presence of their differentiated muscle progeny with increased NF-κB signalling seemed to negatively impact the stem cells and indeed blocking NF-κB specifically in aged muscle fibres improved satellite cell function (Oh *et al.*, 2016). Hence, age-associated non-cell autonomous impacts on satellite cells may also contribute to muscle aberrance in normal ageing and disease.

Muscle-specific inactivation of NF-κB failed to ameliorate loss of muscle mass and neuromuscular function in aged muscle-specific inhibition of NF-κB through expression of IκBα super repressor (MISR) mice (Zhang *et al.*, 2017). Moreover, NF-κB inhibition altered the expression of genes associated with muscle growth and NMJ function and caused accelerated early differentiation *in vitro* (Zhang *et al.*, 2017). This highlights the key role of tightly regulating NF-κB in order to prevent muscle aberrance with ageing. Indeed, NF-κB alterations in various cell types are also implicated in the pathogenesis of ALS (Frakes *et al.*, 2014).

A number of extrinsic signalling pathways (Wnt, TGFβ, Notch, FGF) have been noted to interact closely with ageing satellite cells, with key implications for the regenerative capacity of these cells (Chakkalakal and Brack, 2012). Indeed, Notch activity drops whereas TGFβ and pSmad3 increase in old muscle, inducing a loss of regenerative capacity (as confirmed by three different Smad3-targeted small hairpin RNAs restoring markers to youthful levels in satellite cells and enhancing myogenesis in old muscle) (Carlson *et al.*, 2008). Evidence for the impact of the muscle niche also comes from studies of heterochronic parabiosis, which unite the circulatory systems of aged and young animals, with elderly tissues exposed to youth serum systemic factors. By separating young and aged contributions *in vivo* via GFP reporter labelling, notably, the native aged satellite cells were reactivated and enhanced myogenesis occurred post-injury (Conboy *et al.*, 2005). Delta upregulation, indicative of Notch activity, was restored with exposure to young serum (Conboy *et al.*, 2005). Growth differentiation factor 11 (GDF11) has been implicated as a key circulating rejuvenating factor, restoring structural and even functional deficits in aged mice (Sinha *et al.*, 2014). Muscle transplantation between old and young rats revealed that old to young transplants had greater mass, maximum force and resembled young-young autografts histologically (Carlson and Faulkner, 1989), adding yet more support to the key role of the muscle niche in ageing. A less permissive and poorly sustainable aged muscle environment might prove vulnerable to disease-specific mechanisms, such as those in ALS.

Muscle mitochondrial function decreases with ageing, with wild-type mice showing decreased oxygen consumption rates and increased production of reactive oxygen species (ROS) as they age (Valentine *et al.*, 2018). Autophagy, the lysosome-mediated process by which various cytosolic components are degraded, was diminished in muscle obtained from elderly sedentary patients, and muscle-specific knockout of autophagy-associated ATG7 in mice enhanced muscle atrophy, inflammation, abnormal structure and reduced life expectancy in this model (Carnio *et al.*, 2014). Inhibition of autophagy also increased mitochondria frequency, size and structural aberrance, leading to oxidative stress and ROS, which in turn disturbs interaction between actin and myosin and force generation (Carnio *et al.*, 2014). Old (29 months) male rats showed a maladaptive endoplasmic reticulum (ER) stress response on hindlimb reloading following 14 days of unloading (which had caused disuse-induced atrophy and deficits in force generation) (Baehr *et al.*, 2016). Hence, ER and oxidative stress, mitochondrial dysfunction and autophagy also play key roles in muscle ageing, and indeed, all of these pathways are also implicated in ALS pathogenesis (reviewed in Loeffler *et al.*, 2016; Van Damme *et al.*, 2017).

With the abovementioned mechanisms of normal muscle ageing sharing associations with the pathophysiology of sarcopenia, it is important to consider the role of age-related skeletal muscle perturbations in other diseases such as ALS. With muscle intimately structurally and functionally linked with lower motor neurons, it is possible that defective aged skeletal muscle fails to fulfil its role in the complex relationship, thereby contributing to disease. Indeed, it is at the level of the NMJ where skeletal muscle ageing may play its largest role in ALS. Skeletal muscle expressed FGFBP1, found to be a key protective factor to preserve NMJ integrity, was reduced in both normal ageing and ALS (SOD1^G93A^ mice) (Taetzsch *et al.*, 2017), suggesting a common pathological mechanism between the two. Hence, neuromuscular structural and functional consequences result from the effects of ageing at the level of the skeletal muscle, with potential mechanistic overlaps with ALS.

### Ageing of astrocytes

With non-neuronal cells matching neuronal numbers in the human brain (Azevedo *et al.*, 2009), astrocytes, the most abundant of the CNS glial cells, perform an array of functions fundamental in development and adulthood including synaptogenesis and synaptic elimination, neurotransmitter recycling, blood–brain barrier maintenance and supporting neuronal survival (reviewed in Vasile *et al.*, 2017). With a non-cell autonomous contribution to neurodegenerative disease pathogenesis now widely accepted over the traditional ‘neuron centric’ model, astrocytes have emerged as vital disease players in ALS, with both toxic gain-of-function (Nagai *et al.*, 2007) and loss of neuronal support implicated (Das and Svendsen, 2015; Tyzack *et al.*, 2017). Interestingly, there were a number of similarities between 150 day end-stage SOD1 overexpressing astrocytes and 300 day wild-type aged astrocytes with analysis of growth rates, molecular profiles, markers of senescence and motor neuron survival revealing parallels between ALS and aged wild-type samples (Das and Svendsen, 2015). This indicated that the SOD1 mutant ALS astrocytes were displaying the effects of normal ageing at an accelerated rate (Das and Svendsen, 2015). Indeed, astrocytes undergo significant age-associated alterations, which affect their ability to interact with surrounding cells and consequently their vital functions in the CNS. If astrocytes in ALS are a pathologically hastened form of their normally aged counterparts, a true understanding of astrocyte ageing will provide insight into not only the mechanisms behind age-related neurological decline, but also ALS. This is discussed below.

Astrocytes reacting to injury segregate into two groups dependent on mechanisms of injury, as revealed by transcriptomic analysis (Zamanian *et al.*, 2012). Astrocytes subjected to inflammatory stimuli such as lipopolysaccharide (LPS) adopt an A1 phenotype, and those exposed to ischaemia develop an A2 phenotype, with the former upregulating genes involved in synaptic elimination (e.g. complement cascade), and the latter upregulating neurotrophic, reparative and survival promoting genes (e.g. thrombospondins) (reviewed in Liddelow and Barres, 2017).

Astrocytes in ALS and a number of other neurodegenerative diseases possess an A1 reactive phenotype (Clarke *et al.*, 2018). Aged (2 years) mouse astrocytes from an array of brain regions upregulated more A1 reactive genes (including the complement factor C3) than A2 reactive genes, indicating that normal ageing is associated with the more deleterious A1 astrocytic phenotype (Clarke *et al.*, 2018). Indeed, promotion of complement regulated synaptic elimination by normally aged A1 astrocytes may make the brain more vulnerable to neurodegenerative diseases (Clarke *et al.*, 2018).

Alterations in astrocytes with age render them more susceptible to insult. Pure oxidative stress via hydrogen peroxide exposure and mixed stressors (including oxidative stress) in glucose with or without oxygen deprivation affected primary mouse astrocytes matured *in vitro* more than their young counterparts, indicating disruption in the balance between synthesis and scavenging of reactive oxygen species in older astrocytes (Papadopoulos *et al.*, 1998). Indeed, three key antioxidant species, namely glutathione, catalase and SOD were maintained or even elevated in older astroglia, suggesting alternative mechanisms behind the greater injury in these cells (Papadopoulos *et al.*, 1998). Iron, which catalyses free radical synthesis, was increased in aged astrocytes (Papadopoulos *et al.*, 1998). The enhanced vulnerability of aged astrocytes to oxidative stress may play a key role in disease, with oxidative stress playing an important role in ALS pathogenesis (reviewed in Barber and Shaw, 2010).

In turn, primary astrocyte cultures subjected to oxidative stress (hydrogen peroxide) develop a senescent phenotype, also achieved by other stressors (proteasome inhibition via lactacystin-2 and extensive cellular replication) (Bitto *et al.*, 2010). Stressed cells acquired characteristic morphological features of senescence, cell cycle arrest and expressed senescence-associated markers including β-galactosidase, p16, p21 and p53 (Bitto *et al.*, 2010). Replicative senescence was also seen, with associated reductions in telomere length and G1 cell cycle arrest (Bitto *et al.*, 2010). Given the abovementioned susceptibility of astrocytes to oxidative and other stress (Papadopoulos *et al.*, 1998; Bitto *et al.*, 2010) in normal ageing, the development of their senescent phenotype may carry a range of functional defects which ultimately lead to their failure to support themselves and neurons in ageing and disease. Transcriptomic analysis of multiple regions within aged murine brains and subsequent pathway analysis revealed that cholesterol synthesis was downregulated in aged astrocytes (Boisvert *et al.*, 2018). With cholesterol a key constituent of presynaptic vesicle synthesis, neuronal synaptic function could become perturbed as a result of astrocytic ageing (Boisvert *et al.*, 2018). Genes from immune pathways including antigen presentation and the complement cascade, were upregulated, indicating a propensity towards cellular stress and synaptic elimination in aged astrocytes (Boisvert *et al.*, 2018). Transcriptomic analysis also uncovered stark regional heterogeneity in astrocyte expression profiles both within the murine cortex (Boisvert *et al.*, 2018) and between different human post-mortem brain regions (Soreq *et al.*, 2017). In human brains, the most pronounced age-related shifts in astrocyte region-specific genes were identified in the hippocampus and substantia nigra, major sites of pathology in the two most common age-associated neurodegenerative diseases (Alzheimer’s disease and Parkinson’s disease, respectively) (Soreq *et al.*, 2017). The ageing of astrocytes rather than neurons, which show significantly fewer region-specific gene expression changes with age, may therefore underpin regional vulnerability and sites of pathological involvement in neurodegenerative diseases (Soreq *et al.*, 2017). This finding potentially bears significance for ALS, where there is regional and subtype specific vulnerability (reviewed in Nijssen *et al.*, 2017).

Astrocytes possess the key quality of forming intimate interactions with other glial cells in brain physiology. Their interaction with microglia, the immune cells of the CNS, affects microglial branching and distribution (Lana *et al.*, 2019). In ageing, this direct interaction is impaired, with microglial morphology, distribution and ability to efficiently phagocytose disrupted (Lana *et al.*, 2019). The latter could lead to accumulation of toxic proinflammatory cell debris in the CNS (Lana *et al.*, 2019). Key astrocytic interactions with cells in their local environment thereby become perturbed upon ageing, leading to disruption of other cell types in their vicinity via non-cell autonomous mechanisms.

With their sheer number and multiple functional roles, it is unsurprising that astrocytes are heavily relied upon by the human nervous system. Their disruption with normal ageing can therefore have vital knock-on effects on other surrounding cells, such as neurons and microglia, overall leading to a CNS more vulnerable to age-related pathology and neurodegenerative disease.

### Ageing of Schwann cells

Schwann cells adopt various phenotypes dependent on extrinsic cues. Originating from neural crest, immature Schwann cells can either differentiate into non-myelinating or myelinating Schwann cells, the latter via a promyelin Schwann cell intermediate (reviewed in Jessen *et al.*, 2015; Santosa *et al.*, 2018). Indeed, at the NMJ, the peri-synaptic or terminal Schwann cell (TSC) falls within the non-myelinating category and has been implicated in neuromuscular diseases including ALS (reviewed in Santosa *et al.*, 2018). TSCs have been shown to undergo morphological changes in ALS patients, including developing vast cytoplasmic processes (Bruneteau *et al.*, 2015). Moreover, TSCs, which normally juxtapose the NMJ (Fig. 2), are sometimes found to invade the NMJ itself, occupying the space between the presynaptic motor axon terminal and the postsynaptic membrane (termed the synaptic cleft), in turn reducing the surface area for neuromuscular transmission (Bruneteau *et al.*, 2015). Morphological alterations have also been reported in a SOD1^G93A^ mutant model of ALS, with these changes preceding motor terminal degeneration and denervation (Carrasco *et al.*, 2016*b*). More specifically, it was found that TSCs were lost from NMJs with pre-terminal Schwann cell processes taking their place (Carrasco *et al.*, 2016*b*). Additionally, an absence of immunostaining for P75 (post-denervation marker) and S100 (a Schwann cell marker) following experimental denervation suggests that both TSCs and pre-terminal Schwann cells are lost in SOD1^G93A^ mutant mice, hence unable to facilitate reinnervation following denervation (such as in ALS) (Carrasco *et al.*, 2016*a*). Given the vital role of TSCs in maintaining NMJ health and function, and their significance in disease, understanding the impact of ageing on this cell type is essential to truly appreciating their role in ALS pathogenesis. We discuss ageing phenotypes in Schwann cells before subsequently focusing on TSCs.

Neurons of the peripheral nervous system have a remarkable capacity to regenerate, especially when compared to their central counterparts. Integral to this process are Schwann cells, which whether myelinating or non-myelinating, adopt a repair phenotype post nerve injury, regulated by the transcription factor c-Jun (reviewed in Jessen *et al.*, 2015). Regeneration tracks laid by these cells form scaffolds that facilitate axonal reinnervation of their intended targets (reviewed in Jessen *et al.*, 2015). Ageing in Schwann cells is associated with a decline in regenerative capacity (Painter *et al.*, 2014). Indeed, when compared to young mice at 2 months of age, elderly 24-month-old mice had delayed initiation and slower sensory and motor functional recovery, with 12-month-old mice possessing an intermediate capacity (Painter *et al.*, 2014). Furthermore, aged animals receiving young nerve grafts equalled young functional recovery and young animals receiving aged nerve grafts developed a delay in functional restoration (Painter *et al.*, 2014). Genetic analysis revealed that aged animals had downregulated repair function genes, with age-associated decline in growth factor and mitosis genes, and had failed to suppress a myelinating phenotype after injury when compared to their young counterparts (Painter *et al.*, 2014). In aged animals 1 day post nerve injury, c-Jun, the abovementioned regulator of the Schwann cell repair phenotype, only managed one-fifth of the levels achieved in young animals, in line with aged Schwann cell aberrance in dedifferentiation and subsequent failure in functional regeneration (Painter *et al.*, 2014). With ageing impairing Schwann cell facilitated regeneration, neurons may fail to combat damage experienced in both normal ageing and ALS, leading to an enhanced deleterious phenotype.

Dedifferentiated Schwann cells play a role in luring macrophages to the site of axonal damage after injury (Painter *et al.*, 2014). This function too was disrupted in aged animals, with a delay in macrophage recruitment (Painter *et al.*, 2014). Age-related immune dysfunction was also implicated when grafting sections of rat sciatic nerves from 2- to 18-month-old (young-aged) rats and vice versa (aged-young) with young-young and aged-aged graft controls. Both Schwann cells and macrophages play key roles in debris clearance via phagocytosis after injury (Scheib and Hoke, 2016). Indeed, there was more debris in aged-aged controls compared to young-young grafted animals, with young-aged and aged-young grafts displaying intermediate levels. Hence, as cells involved in debris clearance (Schwann cells and immune macrophages) age, their phagocytotic capacity diminishes, a finding replicated *in vitro* for both cell types (Scheib and Hoke, 2016).

It has been long noted that Schwann cell ultrastructural abnormalities accompany ageing in rat peripheral nerves (Thomas *et al.*, 1980). Schwann cells in aged rats developed a phenotype with extended attenuated processes projecting from adaxonal Schwann cell into the axon, in turn compartmentalizing the axon length into small sections, appearing ‘honeycombed’ (Thomas *et al.*, 1980). Intracytoplasmic inclusions were also noted (Thomas *et al.*, 1980). The presence of disproportionately thin myelin sheaths around some axons also indicated remyelination to be present (Thomas *et al.*, 1980). A reduced myelin diameter was also noted in aged C57BL/6 mice, alongside alterations to essential myelin-related proteins including increased carbonylation and reduced protein expression of PMP22 in sciatic nerves (Hamilton *et al.*, 2016). We speculate that structurally aberrant aged Schwann cells may not be able to function optimally and support neurons, which then may potentially allow disease mechanisms, such as those in ALS, to thrive in an already vulnerable environment.

TSCs in aged wild-type mice showed numerical decline, with a progressively lower proportion of NMJs possessing TSCs between 14 and 33 months of age (100% NMJs had TSCs at 9 months of age) (Snyder-Warwick *et al.*, 2018). This loss was accompanied by structural changes in the remaining TSCs, which displayed thinner processes and irregular TSC bodies with heterogeneous S100 staining (Snyder-Warwick *et al.*, 2018). Brain-specific overexpression of SIRT1, implicated in mammalian ageing, enhanced the number of TSC processes and bodies compared to age-matched controls, with a higher proportion of NMJs possessing TSCs in, altogether, a more youthful phenotype (Snyder-Warwick *et al.*, 2018). Additionally, the knockdown of SIRT1 specific to the dorsomedial hypothalamus led to excessively large TSC bodies that frequently resided outside the NMJ, as well as fewer TSCs per NMJ (Snyder-Warwick *et al.*, 2018). Although aberrance was not identical in knockdown and aged wild-type animals, both showed increased frequency of TSC abnormalities, with the knockdown potentially a ‘more aged’ phenotype (Snyder-Warwick *et al.*, 2018). Their imperative roles in sustaining optimal NMJ function implicate TSCs as being a highly relevant cellular candidate linking ageing and ALS.

## Discussion

### Ageing and amyotrophic lateral sclerosis

In this review, we have discussed the effects of normal ageing on the individual cellular components of the lower motor unit, and their potential mechanistic link with ALS (see Table 1 for an overall summary of key similarities between ageing and ALS). With disruption of the NMJ clearly implicated in ALS pathogenesis (Fischer *et al.*, 2004; Wong and Martin, 2010; Shahidullah *et al.*, 2013; Chand *et al.*, 2018) and age-related changes to both individual cellular constituents (discussed above) and the NMJ described (reviewed in Cappello and Francolini, 2017), there is a real role for the unification of ageing and ALS research in order to gain true mechanistic insight into this universally fatal and devastating disease.

**Table 1:** Summary: the interplay between ageing and ALS

Lower motor unit cell type	Normal ageing	Key references	Amyotrophic lateral sclerosis	Key references
**Motor neurons**	Reduction in motor neuron number with ageing Loss of synaptic inputs Electrical abnormalities ‘Senescence like’ alterations Lipofuscin accumulation Mitochondrial aberrance Age-dependency in motor phenotypes and MN degeneration	Tomlinson and Irving, 1977 Maxwell *et al.*, 2018 Moldovan *et al.*, 2016 Ohashi *et al.*, 2018 Moreno-Garcia *et al.*, 2018^a^ Rygiel *et al.*, 2014 Azpurua *et al.*, 2018 Srivastava *et al.*, 2012	Loss of MNs in ALS (degeneration) Loss of synaptic inputs Excitotoxicity Cytoskeletal changes RNA metabolism alterations Mitochondrial aberrance Axonal transport defects Ageing risk factor for MN degeneration in ALS	Vaughan *et al.*, 2015 Van Damme *et al.*, 2017 ^a^ Azpurua *et al.*, 2018 de Waard *et al.*, 2010
**Skeletal muscle**	Sarcopenia: age-associated muscle weakness/wasting Satellite cells: loss of regenerative capacity; poor proliferation and self-renewal; senescence Altered skeletal muscle niche/environment NF-κB implications Mitochondrial dysfunction, oxidative stress, autophagy alterations, ER stress FGFBP1 maintains NMJ in ageing	Marzetti *et al.*, 2017 ^a^ Blanc *et al.*, 2016 Sousa-Victor *et al.*, 2014*b* Conboy *et al.*, 2005 Oh *et al.*, 2016 Valentine *et al.*, 2018 Carnio *et al.*, 2014 Baehr *et al.*, 2016 Taetzsch *et al.*, 2017	ALS: early muscle symptoms-weakness; wasting Muscle specific expression of SOD1 → MN degeneration (die-back hypothesis) Satellite cells: loss of regenerative capacity NF-κB implications Mitochondrial dysfunction, oxidative/ER stress and autophagy defects = proposed ALS mechanisms FGFBP1 maintains NMJ in an ALS model	Hobson and McDermott, 2016 ^a^ Wong and Martin, 2010 Scaramozza *et al.*, 2014 Frakes *et al.*, 2014 Barber and Shaw, 2010 ^a^ Van Damme *et al.*, 2017 ^a^ Taetzsch *et al.*, 2017
**Astrocytes**	Ageing upregulates A1 reactive genes > A2 Aged ACs are vulnerable to oxidative stress Senescence Loss of AC neuronal support functions with ageing (e.g. cholesterol synthesis) Age-associated regional heterogeneity in AC expression Disrupted interaction with microglia-proinflammatory	Clarke *et al.*, 2018 Papadopoulos *et al.*, 1998 Bitto *et al.*, 2010 Boisvert *et al.*, 2018 Soreq *et al.*, 2017 Lana *et al.*, 2019	A1 AC phenotype in ALS Oxidative stress is a proposed ALS mechanism ACs in ALS: evidence for toxic gain-of-function and loss of homeostatic function mechanisms Differential regional vulnerability to neurodegeneration and pathology might relate AC expression changes with ageing Neuroinflammation is a proposed ALS mechanism	Clarke *et al.*, 2018 Barber and Shaw, 2010 ^a^ Nagai *et al.*, 2007 Tyzack *et al.*, 2017 Soreq *et al.*, 2017 Van Damme *et al.*, 2017 ^a^
**Schwann cells**	Disrupted macrophage interaction and phagocytosis Loss of Schwann cell dedifferentiation potential and regenerative capacity with ageing Disrupted Schwann cell structure with ageing Terminal Schwann cell numerical decline with ageing, with remaining TSCs structurally aberrant	Scheib and Hoke, 2016 Painter *et al.*, 2014 Thomas *et al.*, 1980 Snyder-Warwick *et al.*, 2018	TSC morphological, structural and numerical alterations are implicated in ALS Loss of Schwann cell regenerative capacity in ALS	Bruneteau *et al.*, 2015 Carrasco *et al.*, 2016*b* Carrasco *et al.*, 2016*a*

Refer to the ‘Discussion’ section for further evidence supporting the interplay between ageing and ALS. AC = astrocyte; MN = motor neuron.

Individual cellular components of the lower motor unit (Fig. 2) undergo an array of changes in both normal ageing and ALS, a number of which are summarized above but discussed in detail in the text. Indeed, careful interrogation of overlapping molecular/cellular phenotypic alterations in ageing and ALS might reveal key insights into the interplay between this ubiquitous physiological phenomenon and the rapidly progressive, universally fatal age-associated neurodegenerative disease. ^a^Review articles.

Several studies have more directly investigated the link between normal ageing and ALS. Transcriptomic analysis of iPSC-derived spinal motor neurons, foetal and adult spinal tissues suggested that gene expression networks involved in spinal motor neuron maturation and ageing are also implicated in sporadic ALS (Ho *et al.*, 2016). Levels of SIRT1 decrease during murine ageing, and knockout in motor neurons revealed less NMJ innervation with age, suggesting a role for SIRT1 in preventing NMJ damage with age (Herskovits *et al.*, 2018). Transcriptomic analysis of SOD1^G93A^ murine spinal cords identified an overlap between ageing and ALS (90% of aged spinal cord transcripts upregulated in ALS), with inflammation and immune system activation being key pathways (Herskovits *et al.*, 2018). Interestingly, overexpression of SIRT1 in motor neurons delayed ALS disease progression in SOD1^G93A^ mice (Herskovits *et al.*, 2018), showing that interventions targeting ageing can in fact benefit ALS.

Telomere shortening is a hallmark of normal cellular ageing. SOD1^G93A^ mice crossed with telomerase knockout mice showed earlier disease onset, shortened life expectancy and an overall enhanced pathological phenotype (Linkus *et al.*, 2016), indicating a role for telomere dysfunction in ALS. Moreover, in patients with sporadic ALS, human telomerase reverse transcriptase (hTERT), a component of the telomerase enzyme, was lower in post-mortem spinal cords, a result replicated in leucocytes from patient blood samples compared to healthy control subjects (De Felice *et al.*, 2014). Indeed, telomere length was significantly reduced in patients too (De Felice *et al.*, 2014). Altogether, given the neuroprotective roles of telomerase in combatting cellular stresses, alterations of this enzyme with ageing may lead to vulnerability of neurons and contribute to ALS pathology (De Felice *et al.*, 2014).

Day 32 human iPSC-derived TDP-43 mutant motor neurons showed enhanced vulnerability and neurodegeneration compared to their Day 5 counterparts and wild-type controls (Kreiter *et al.*, 2018). Alterations to cytoskeletal morphology and axonal mitochondria and lysosomes were noted, with size, shape, and organelle motility modified (Kreiter *et al.*, 2018). Interestingly, these mechanisms of motor neuronal degeneration were independent of the TDP-43 cytoplasmic aggregation ALS pathological hallmark (Kreiter *et al.*, 2018), indicating that looking at ageing and ALS together can uncover novel pathological mechanisms and potentially yield future therapeutic targets.

The question remains as to why certain individuals are selectively vulnerable to ALS, whilst others grow old without acquiring ALS or other age-associated neurodegenerative diseases. An interindividual heterogeneity in susceptibility to ageing might contribute to the explanation, with recent evidence suggesting that certain individuals age better and others worse than expected, termed Delta ageing (Rhinn and Abeliovich, 2017) (Fig. 1). Additionally, TMEM106B and progranulin were identified as accelerators of ageing (Rhinn and Abeliovich, 2017), indicating that such factors might determine the effects of ageing on an organism level. Differential rates of ageing were also noted at a cellular level, notably, within a single anatomical region (Maxwell *et al.*, 2018). Alpha motor neurons, which showed no difference in number and size on ageing, were found to have greatly varied amounts of lipofuscin accumulation, reflecting subcellular changes (Maxwell *et al.*, 2018). Differential susceptibility to ageing was noted amongst old mouse NMJs in an array of muscles, falling into three categories: muscles susceptible in early ageing, muscles with a delayed response to ageing, and muscles resistant to the effects of ageing, such as extraocular muscles, which are also known to be spared in ALS (Valdez *et al.*, 2012). Comparison to SOD1^G93A^ NMJs in these muscles revealed similar susceptibility and phenotypes in ALS and ageing (Valdez *et al.*, 2012). Despite a consensus that TDP-43 pathology is absent in SOD1-ALS (Mackenzie *et al.*, 2007), cytoplasmic TDP-43 aggregates were found in spinal motor neurons in old mice and SOD1^G93A^, showing a stark overlap in pathology between ageing and ALS (Valdez *et al.*, 2012). Hence, with ageing affecting cells at different rates, it is possible that ‘Delta ageing’ occurs on both an organism level and cellular level, maybe somewhat accounting for varied susceptibility to ALS between and within individuals.

With mechanisms of ageing and ALS showing a number of parallels, there is an unmet requirement to integrate the two fields of research. An approach is to age existing models of ALS so that they faithfully recapitulate the age-associated human presentation of the disease. Integrative modelling is the optimal approach for validation of key findings and for providing best evidence for a mechanistic link between ageing and ALS. Specifically, the unification of *in vitro* and *in vivo*, animal and human models of ALS and ageing, alongside post-mortem tissue, each with their own benefits and drawbacks (Table 2), is key to achieve high fidelity conclusions. Human iPSCs provide a patient and human-specific model of disease, with familial and sporadic ALS patient iPSC-derived neurons recapitulating a number of disease-specific phenotypes (Hall *et al.*, 2017; Tyzack *et al.*, 2017, 2019; Fujimori *et al.*, 2018; Luisier *et al.*, 2018; Simone *et al.*, 2018; reviewed in Ziff and Patani, 2019). They therefore provide a useful and simplified model of human neurodegenerative disease. However, during reprogramming, human iPSCs obtain foetal age profiles, resetting age-related genetic, epigenetic and phenotypic signatures of their donors (Miller *et al.*, 2013; Mertens *et al.*, 2015; Ho *et al.*, 2016). It is thereby plausible that human iPSC studies are picking up early disease changes rather than relevant later disease phenotypes that require ageing, highlighting the need for adding age to existing ALS models.

**Table 2:** Integrative modelling

Animal models (*in vivo*)		Cell models (*in vitro*)		Post-mortem tissue	

Benefits	Limitations	Benefits	Limitations	Benefits	Limitations
		**Primary cultures**			
Can model functionality on an organism level 3D model Transgenic models can capture molecular, cellular and functional phenotypes of disease Allows disease understanding in context of full complement of other cell types Measures such as life expectancy and the development of pathology over time are valuable Ageing can be easily recapitulated with longitudinal observation in animals Some animals have human resembling anatomy and prove better models of human disease e.g. primates A number of *in vivo* models for the same disease are often available for cross validation	Some transgenic models overexpress genes to supraphysiological levels, losing fidelity Vast differences in anatomy, lifespan and physiology compared to humans Development and disease mechanisms can differ amongst different species Complex *in vivo* environment might introduce a number of confounders Species differences make it difficult to form reliable conclusions for human disease Longitudinal ageing studies are time consuming (rodents must be maintained for months to years and higher order animal models e.g. primates, will take even longer to reach old age) **Lack of translational success** Cannot model sporadic disease	High availability from *in vivo* animal models but non-proliferative and do not self-renew (neurons) Maintain *in vivo* cellular interactions *in vitro* Can study chronological pathology development	Faster results and lower maintenance requirements than human iPSCs 2D model Cannot obtain primary human neurons until post-mortem **Lack of translational success**	Human and patient specific model Capture of pathological hallmarks of disease and localization to anatomical regions Ability to observe pathology temporality via samples of patients of various age: require large sample sizes (Braak studies) Account for human cellular complexity Ability to visualize histopathological hallmarks Ability to reliably isolate anatomical regions Can compare cell type-specific vulnerability by identifying which cell types are most affected by pathology Can model sporadic disease	Variations in post-mortem delay - difficult to account for Single snapshot of end-stage disease - terminal model fails to capture initiating pathology Tissue alterations *ex vivo* before analysis: samples may not be exposed to identical environments e.g. temperatures as might be obtained at significantly different times Limited tissue availability - reliance on organ donors Patient comorbidity or cause of death might confound conclusions Difficulty in study of rare diseases due to lack of availability of patient samples **Lack of translational success**

		**Human iPSCs**			
		Human and patient specific model Co-culture allows study of non-cell autonomous disease mechanisms Capacity to self-renew and continue proliferation Retains patient-specific mutations for disease study Ability to visualize first signs of pathology Can direct differentiation to a number of fates 3D models exist Can model sporadic disease	Foetal age profile Simplified model that might not reflect *in vivo* cellular complexity Require developmentally rationalized directed differentiation paradigms to yield pure cell types efficiently High cost and time demand 2D model **Lack of translational success**		

A range of ALS models exist which recapitulate molecular, cellular and functional phenotypes of the disease; however, these models are yet to provide patients with therapies that significantly enhance life quality or expectancy. Each method of studying ALS has benefits and limitations (reviewed in Serio and Patani, 2018), and all have capacity to incorporate ageing, thereby allowing better representation of age-associated neurodegenerative diseases such as ALS. Cross-validation of results by integrating the various methods allows acquisition of reliable, high fidelity results. The fusion of ageing into existing *in vivo* and *in vitro* ALS models and post-mortem tissue and cross-validation of results via all approaches will ultimately benefit bench to bedside translation and in turn, patient lifespan and healthspan.

*In vitro*, a number of approaches have been taken to age cells so that they better replicate *in vivo* disease pathogenesis. The small molecule inhibitor of telomerase, BIBR1532, shortened telomeres in human iPSC-derived midbrain dopaminergic neurons (Vera *et al.*, 2016), capturing not only age-related but also disease-specific phenotypes in a cell type-specific manner (Vera *et al.*, 2016). Overexpression of progerin, the mutant protein underlying Hutchinson-Gilford progeria syndrome (characterized by premature ageing), revealed an enhanced disease phenotype in the aged model, with aged grafts also failing to provide functional recovery in a mouse model (Miller *et al.*, 2013). Genotype-specific phenotypes (absent from all controls) were noted with progerin overexpression (Miller *et al.*, 2013), indicating that certain genotype-specific phenotypes might only be revealed in an aged system. Despite their focus on Parkinson’s disease, these studies fortify the link between normal ageing and neurodegenerative diseases. Indeed, similar approaches are required to delineate cell type-specific features of ageing in ALS.

**Figure 3:**
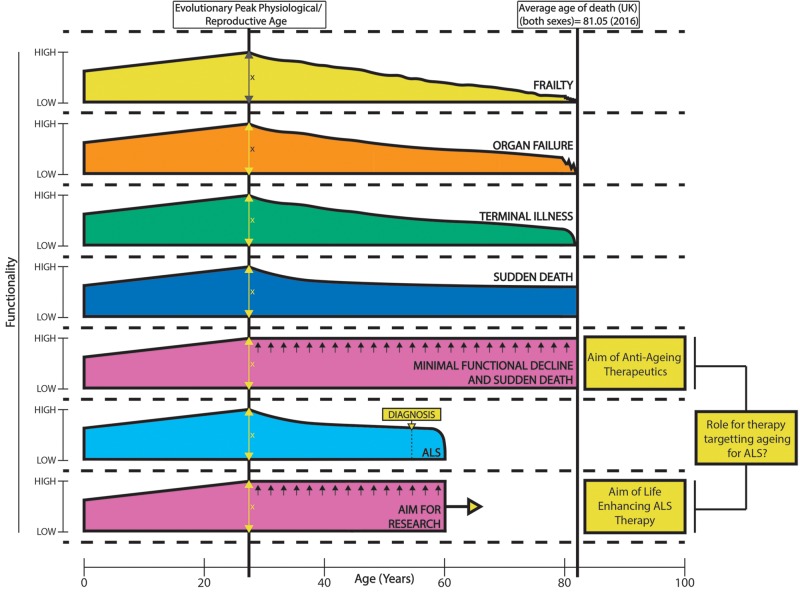
**Healthspan versus lifespan: ageing and ALS.** Patient functionality alters with age. There is an increase in functionality from birth to optimum reproductive age where, evolutionarily, humans reach peak performance to give best chance of survival on a species level. From then, there is a gradual decline in functionality that can lead to disability once a certain threshold is passed. In ALS, this functional decline is particularly pronounced, with end of life trajectory of terminal illness and death at a much younger age. A variety of end of life trajectories exist, leading to significant disability before death (when compared to sudden death where there is no further functional decline) (Lunney *et al.*, 2003). Functionality is a key component of quality of life, so while lifespan or longevity is seen on the *x*-axis, healthspan (years spent in good health/quality of life/functionality) is seen on the *y*-axis. The aim of therapeutics in ageing (Crimmins, 2015; Olshansky, 2018) and ALS research is to maximize healthspan and minimize functional decline and disability.

Bypassing the pluripotent state by direct transdifferentiation of donor fibroblasts to induced neurons maintains age-associated expression profiles from donors, in contrast to reprogramming to an iPSC state (Mertens *et al.*, 2015). A comparison of expression profiles from ageing fibroblasts, induced neurons and ageing human cortical tissue revealed RANBP17 (a nuclear pore associated transport protein) as an ageing factor (Mertens *et al.*, 2015). Brain levels of the protein decreased with ageing as did amounts in mature induced neurons, where reduced RANBP17 induced a functional age-associated phenotype (Mertens *et al.*, 2015). Knockdown of RANBP17 via short hairpin RNAs caused age-associated alterations to young fibroblast transcriptomes (Mertens *et al.*, 2015). Transdifferentiation and formation of induced neurons therefore provides a patient- and human-specific *in vitro* model that maintains age-related genetic signatures, which iPSCs do not. Recently, heterochromatin protein 1 binding protein 3 (HP1BP3) was identified as a mediator of ageing, with hippocampal knockdown by virally introduced short hairpin RNA causing working memory and contextual fear memory aberrance (cognitive disruption), transcriptomic alterations, and reducing neuronal excitability and synaptic plasticity (Neuner *et al.*, 2018). Notably, there was a large overlap between downregulated genes in knockdown conditions and genes downregulated in human frontal cortex ageing, suggesting HP1BP3 is a key regulator in ageing, with age-related alterations at molecular, cellular and even functional levels noted *in vivo* (Neuner *et al.*, 2018). The integration of ageing and ALS research via more relevant patient models will ultimately provide more reliable therapeutic interventions for patients. Indeed the failure of translation thus far, emphasized by the fact that Riluzole is the only UK approved pharmaceutical life enhancing therapy for ALS patients, might resemble a failure to accurately model the disease (Johnson, 2015). High fidelity models that account for age-related cell type-specific effects will lead to therapeutics that might in turn enhance patient healthspan (length of time living in optimal health) and lifespan (life expectancy). Indeed, the aim of both ALS (rapid progressive functional decline) and ageing therapeutics is to allow patients to live longer in optimum health (enhance healthspan), so that quality of life is maximized (Fig. 3). An integration of research into ageing and ALS thereby unlocks the potential for therapeutic advancement in both fields.

## Concluding remarks

In this review, we have discussed the potential role of cell type-specific ageing in ALS. We critically review evidence for the overlap between ageing and ALS on molecular, cellular and functional levels, suggesting that normal ageing could have an important contribution to ALS, likely alongside other genetic, lifestyle and environmental factors. With accumulating literature for mechanistic parallels between normal ageing and ALS, the unification of the two research fields, development of ALS models incorporating ageing and common aim of enhanced patient healthspan will ultimately provide life—quality and quantity—enhancing therapy for patients.

## Funding

V.A.P. is funded by the Rosetrees Trust [548644] and the University College London MBPhD Programme. R.P. holds an MRC Senior Clinical Fellowship [MR/S006591/1].

## Competing interests

The authors report no competing interests.

## Abbreviations

ALS =: amyotrophic lateral sclerosis
iPSC =: induced pluripotent stem cell
NMJ =: neuromuscular junction
TSC =: terminal Schwann cell
